# Crystal structure of a mixed solvated form of amoxapine acetate

**DOI:** 10.1107/S2056989014028096

**Published:** 2015-01-10

**Authors:** Rajni M. Bhardwaj, Vishal Raval, Iain D. H. Oswald, Alastair J. Florence

**Affiliations:** a Strathclyde Institute of Pharmacy and Biomedical Sciences, University of Strathclyde, 161 Cathedral Street, Glasgow G4 0RE, Scotland

**Keywords:** crystal structure, amoxapine, oxazepine, mixed solvate, hydrogen bonding.

## Abstract

The mixed solvated salt 4-(2-chloro­dibenzo[*b*,*f*][1,4]oxazepin-11-yl)piperazin-1-ium acetate–acetic acid–cyclo­hexane (2/2/1), C_17_H_17_ClN_3_O^+^·C_2_H_3_O_2_^−^·C_2_H_4_O_2_·0.5C_6_H_12_, crystallizes with one mol­ecule of protonated amoxapine (AXPN), an acetate anion and a mol­ecule of acetic acid together with half a mol­ecule of cyclo­hexane. In the centrosymmetric crystal, both enanti­omers of the protonated AXPN mol­ecule stack alternatively along [001]. Acetate anions connect the AXPN cations through N—H⋯O hydrogen bonding in the [010] direction, creating a sheet lying parallel to (100). The acetic acid mol­ecules are linked to the acetate anions *via* O—H⋯O hydrogen bonds within the sheets. Within the sheets there are also a number of C—H⋯O hydrogen bonds present. The cyclo­hexane solvent mol­ecules occupy the space between the sheets.

## Abstract 2

The mixed solvated salt 4-(2-chloro­dibenzo[*b*,*f*][1,4]oxazepin-11-yl)piperazin-1-ium acetate–acetic acid–cyclo­hexane (2/2/1), crystallizes with one mol­ecule of protonated amoxapine (AXPN), an acetate anion and a mol­ecule of acetic acid together with half a mol­ecule of cyclo­hexane. In the crystal, the various components are linked *via* N—H⋯O and O—H⋯O hydrogen bonds, forming a layered structure with the solvent mol­ecules occupying the spaces between the layers.

## Chemical context   

2-Chloro-11-(piperazin-1-yl)dibenzo[*b*,*f*][1,4]oxazepine (Amox­apine, AXPN) is a benzodiazepine derivative and exhibits anti-depressant properties (Greenbla & Osterber, 1968 ▸) with one reported crystal structure (CSD refcode: AMOXAP; Cosulich & Lovell, 1977 ▸). AXPN acetate acetic acid cyclo­hexane was obtained as a part of a wider investigation that couples experimental crystallization techniques with computational methods in order to obtain a better understanding of the factors underpinning the solid-state structure and diversity of structurally related compounds, *i.e.* olanzapine, clozapine, loxapine and AXPN (Bhardwaj & Florence, 2013 ▸; Bhardwaj, Johnston *et al.*, 2013 ▸; Bhardwaj, Price *et al.*, 2013 ▸). The sample of AXPN acetate acetic acid cyclo­hexane was isolated during an experimental physical form screen of AXPN. The sample was identified as a novel form using multi-sample foil transmission X-ray powder diffraction analysis (Florence *et al.*, 2003 ▸). A suitable sample for single crystal X-ray diffraction analysis was obtained from slow evaporation of a saturated solution of AXPN in a 1:1 molar ratio of acetic acid and cyclo­hexane at room temperature.
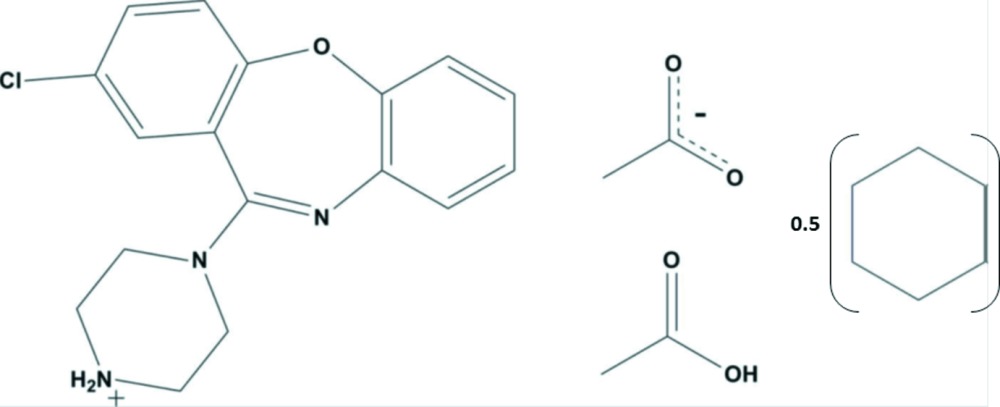


## Structural commentary   

**Figure 1:**
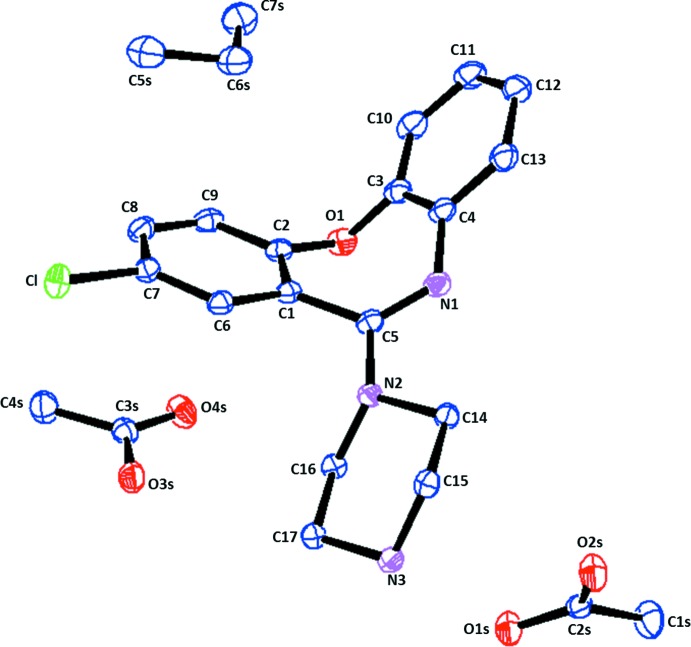
A view of the mol­ecular structure of the asymmetric unit of the title mol­ecular salt, showing the atom labelling. Displacement ellipsoids are drawn at the 50% probability level.

The title compound crystallizes with one mol­ecule of protonated AXPN and an acetate anion each with a mol­ecule of acetic acid and a half mol­ecule of cyclo­hexane (which lies across a center of inversion) as solvent of crystallization in the asymmetric unit (Fig. 1 ▸). The dioxazepine ring of AXPN exists in a puckered conformation between the planes of the benzene rings [the benzene rings fused to the central ring make a dihedral angle of 58.63 (6)°], and the piperazine ring adopts a chair conformation, as observed in the AXPN free base (CSD refcode: AMOXAP; Cosulich and Lovell, 1977 ▸) and structurally related analogues (Bhardwaj & Florence, 2013 ▸; Bhardwaj, Johnston *et al.*, 2013 ▸; Bhardwaj, Price *et al.*, 2013 ▸).

## Supra­molecular features   

**Figure 2:**
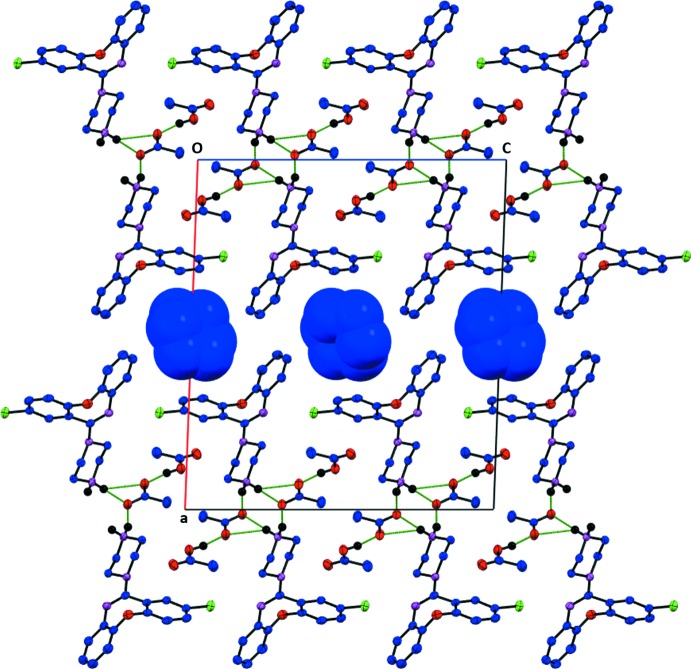
The crystal packing of the title mol­ecular salt, viewed down the *b* axis. The cyclo­hexane mol­ecules are shown as a blue space-fill model. Hydrogen bonds are shown as green lines (see Table 1 ▸ for details; atom colour code: C, N, O, Cl and H are blue, violet, red, green and black, respectively; H atoms not involved in hydrogen bonding have been omitted for clarity).

In the crystal, opposite enanti­omers of protonated AXPN mol­ecules stack along the *c-*axis direction. Each protonated AXPN mol­ecule forms two N—H⋯O hydrogen bonds with two acetate anions, which connect it to an adjacent protonated AXPN mol­ecule along the *b* axis, creating a sheet-like structure parallel to (100); see Fig. 2 ▸ and Table 1 ▸. The acetic acid mol­ecules act as hydrogen-bond donors to acetate anions and are present between the protonated AXPN mol­ecules along the *c*-axis direction. There are also C—H⋯O hydrogen bonds present within the sheets (Table 1 ▸). These sheets stack along the *a* axis and the cyclo­hexane mol­ecules occupy the space between the sheets (Fig. 2 ▸).

## Synthesis and crystallization   

Rod-shaped crystals were grown from a saturated solution of AXPN in a 1:1 molar ratio of acetic acid and cyclo­hexane by isothermal solvent evaporation at 298 K. 

## Refinement   

Crystal data, data collection and structure refinement details are summarized in Table 2 ▸. The N- and O-bound H atoms were located in a difference Fourier map and freely refined. The C-bound H atoms were placed in calculated positions and refined as riding atoms: C—H = 0.95–0.99 Å with *U*_iso_(H) = 1.5*U*_eq_(C) for methyl H atoms and = 1.2*U*_eq_(C) for other H atoms.

## Supplementary Material

Crystal structure: contains datablock(s) I. DOI: 10.1107/S2056989014028096/su5039sup1.cif

Structure factors: contains datablock(s) I. DOI: 10.1107/S2056989014028096/su5039Isup2.hkl

Supporting information file. DOI: 10.1107/S2056989014028096/su5039Isup3.mol

Supporting information file. DOI: 10.1107/S2056989014028096/su5039Isup4.cml

CCDC reference: 1040948

Additional supporting information:  crystallographic information; 3D view; checkCIF report

## Figures and Tables

**Table 1:** Hydrogen-bond geometry (, )

*D*H*A*	*D*H	H*A*	*D* *A*	*D*H*A*
N3H1*N*3O1*S*	0.91(2)	1.86(2)	2.7664(13)	175(2)
O3*S*H1*S*O2*S* ^i^	0.94(2)	1.61(2)	2.5375(13)	171(2)
N3H2*N*3O1*S* ^ii^	0.94(2)	1.82(2)	2.7292(14)	162(1)
C1*S*H1*S*1O3*S* ^ii^	0.96	2.42	3.3778(18)	172
C14H14*A*O1^iii^	0.97	2.59	3.2448(15)	125
C17H17*A*O4*S* ^iii^	0.97	2.32	3.2314(15)	155

Symmetry codes: (i) 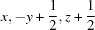; (ii) 
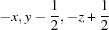
; (iii) 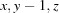.

**Table 2:** Experimental details

Crystal data	
Chemical formula	C_17_H_17_ClN_3_O^+^C_2_H_3_O_2_ C_2_H_4_O_2_0.5C_6_H_12_
*M*_r_	475.96
Crystal system, space group	Monoclinic, *P*2_1_/*c*
Temperature (K)	150
*a*, *b*, *c* ()	21.0726(12), 6.0393(3), 18.6087(10)
()	92.096(2)
*V* (^3^)	2366.6(2)
*Z*	4
Radiation type	Mo *K*
(mm^1^)	0.20
Crystal size (mm)	0.55 0.22 0.11

Data collection	
Diffractometer	Bruker APEXII CCD
Absorption correction	Multi-scan (*SADABS*; Bruker, 2007 ▸)
*T*_min_, *T*_max_	0.647, 0.745
No. of measured, independent and observed [*I* > 2(*I*)] reflections	18828, 4860, 4177
*R*_int_	0.018
(sin /)_max_ (^1^)	0.626

Refinement	
*R*[*F* ^2^ > 2(*F* ^2^)], *wR*(*F* ^2^), *S*	0.030, 0.082, 1.03
No. of reflections	4860
No. of parameters	312
H-atom treatment	H atoms treated by a mixture of independent and constrained refinement
_max_, _min_ (e ^3^)	0.28, 0.22

Computer programs: *APEX2* and *SAINT* (Bruker, 2007 ▸), *SHELXS97* and *SHELXL97* (Sheldrick, 2008 ▸), *Mercury* (Macrae *et al.*, 2008 ▸), *ORTEP-3 for Windows* (Farrugia, 2012 ▸), *enCIFer* (Allen *et al.*, 2004 ▸) and *publCIF* (Westrip, 2010 ▸).

## supplementary crystallographic information

### Crystal data

**Table:**

C_17_H_17_ClN_3_O^+^·C_2_H_3_O_2_^−^·C_2_H_4_O_2_·0.5C_6_H_12_	*F*(000) = 1008
*M**_r_* = 475.96	*D*_x_ = 1.336 Mg m^−^^3^
Monoclinic, *P*2_1_/*c*	Mo *K*α radiation, λ = 0.71073 Å
Hall symbol: -P 2ybc	Cell parameters from 9940 reflections
*a* = 21.0726 (12) Å	θ = 2.9–26.4°
*b* = 6.0393 (3) Å	µ = 0.20 mm^−^^1^
*c* = 18.6087 (10) Å	*T* = 150 K
β = 92.096 (2)°	Rod, colourless
*V* = 2366.6 (2) Å^3^	0.55 × 0.22 × 0.11 mm
*Z* = 4	

### Data collection

**Table:**

Bruker APEXII CCD diffractometer	4860 independent reflections
Radiation source: fine-focus sealed tube	4177 reflections with *I* > 2σ(*I*)
Graphite monochromator	*R*_int_ = 0.018
φ and ω scans	θ_max_ = 26.4°, θ_min_ = 1.9°
Absorption correction: multi-scan (*SADABS*; Bruker, 2007)	*h* = −26→26
*T*_min_ = 0.647, *T*_max_ = 0.745	*k* = −6→7
18828 measured reflections	*l* = −23→21

### Refinement

**Table:**

Refinement on *F*^2^	Primary atom site location: structure-invariant direct methods
Least-squares matrix: full	Secondary atom site location: difference Fourier map
*R*[*F*^2^ > 2σ(*F*^2^)] = 0.030	Hydrogen site location: inferred from neighbouring sites
*wR*(*F*^2^) = 0.082	H atoms treated by a mixture of independent and constrained refinement
*S* = 1.03	*w* = 1/[σ^2^(*F*_o_^2^) + (0.0383*P*)^2^ + 1.089*P*] where *P* = (*F*_o_^2^ + 2*F*_c_^2^)/3
4860 reflections	(Δ/σ)_max_ = 0.001
312 parameters	Δρ_max_ = 0.28 e Å^−^^3^
0 restraints	Δρ_min_ = −0.22 e Å^−^^3^

### Special details

**Table:**

Geometry. All s.u.'s (except the s.u. in the dihedral angle between two l.s. planes) are estimated using the full covariance matrix. The cell s.u.'s are taken into account individually in the estimation of s.u.'s in distances, angles and torsion angles; correlations between s.u.'s in cell parameters are only used when they are defined by crystal symmetry. An approximate (isotropic) treatment of cell s.u.'s is used for estimating s.u.'s involving l.s. planes.
Refinement. Refinement of *F*^2^ against ALL reflections. The weighted *R*-factor *wR* and goodness of fit *S* are based on *F*^2^, conventional *R*-factors *R* are based on *F*, with *F* set to zero for negative *F*^2^. The threshold expression of *F*^2^ > 2σ(*F*^2^) is used only for calculating *R*-factors(gt) *etc*. and is not relevant to the choice of reflections for refinement. *R*-factors based on *F*^2^ are statistically about twice as large as those based on *F*, and *R*- factors based on ALL data will be even larger.

### Fractional atomic coordinates and isotropic or equivalent isotropic displacement parameters (Å^2^)

**Table:**

	*x*	*y*	*z*	*U*_iso_*/*U*_eq_	
H2N3	0.0503 (7)	−0.014 (3)	0.3171 (8)	0.029 (4)*	
H1N3	0.0584 (7)	0.167 (3)	0.2658 (9)	0.025 (4)*	
H1S	0.1043 (10)	0.564 (4)	0.5635 (12)	0.064 (6)*	
Cl	0.276361 (16)	0.39554 (6)	0.594267 (17)	0.02772 (10)	
O1	0.30157 (4)	0.90957 (15)	0.32837 (5)	0.0225 (2)	
O2S	0.07221 (4)	0.03016 (16)	0.13430 (5)	0.0259 (2)	
O1S	0.01425 (4)	0.28471 (16)	0.18632 (5)	0.0234 (2)	
N3	0.07793 (5)	0.09975 (18)	0.30430 (6)	0.0161 (2)	
N2	0.19325 (5)	0.34168 (17)	0.31916 (5)	0.0160 (2)	
N1	0.27645 (5)	0.48819 (18)	0.25813 (6)	0.0196 (2)	
C4	0.33285 (6)	0.6088 (2)	0.25098 (7)	0.0198 (3)	
C6	0.25997 (5)	0.4584 (2)	0.45099 (6)	0.0177 (2)	
H6	0.2404	0.3204	0.4501	0.021*	
C2S	0.03703 (5)	0.1974 (2)	0.13128 (6)	0.0175 (2)	
C5	0.24619 (5)	0.4789 (2)	0.31684 (6)	0.0170 (2)	
C2	0.29573 (5)	0.7852 (2)	0.39086 (7)	0.0183 (3)	
C8	0.31125 (6)	0.7556 (2)	0.51865 (7)	0.0228 (3)	
H8	0.3256	0.8140	0.5625	0.027*	
C15	0.13972 (6)	0.0041 (2)	0.28291 (6)	0.0167 (2)	
H15A	0.1326	−0.1006	0.2439	0.020*	
H15B	0.1596	−0.0741	0.3233	0.020*	
C1	0.26655 (5)	0.5783 (2)	0.38728 (7)	0.0168 (2)	
C17	0.08763 (5)	0.2614 (2)	0.36432 (6)	0.0169 (2)	
H17A	0.1044	0.1855	0.4068	0.020*	
H17B	0.0473	0.3278	0.3757	0.020*	
C7	0.28300 (6)	0.5483 (2)	0.51529 (7)	0.0200 (3)	
C9	0.31772 (6)	0.8743 (2)	0.45570 (7)	0.0219 (3)	
H9	0.3368	1.0133	0.4569	0.026*	
C16	0.13360 (5)	0.4404 (2)	0.34257 (7)	0.0164 (2)	
H16A	0.1145	0.5276	0.3037	0.020*	
H16B	0.1425	0.5384	0.3830	0.020*	
C10	0.39883 (6)	0.9372 (2)	0.26691 (7)	0.0264 (3)	
H10	0.4059	1.0745	0.2884	0.032*	
C3	0.34532 (6)	0.8156 (2)	0.28257 (7)	0.0208 (3)	
C13	0.37702 (6)	0.5278 (2)	0.20305 (7)	0.0234 (3)	
H13	0.3701	0.3911	0.1811	0.028*	
C14	0.18287 (6)	0.1887 (2)	0.25899 (6)	0.0167 (2)	
H14A	0.2231	0.1280	0.2446	0.020*	
H14B	0.1634	0.2660	0.2182	0.020*	
C12	0.43094 (6)	0.6479 (3)	0.18768 (7)	0.0275 (3)	
H12	0.4600	0.5904	0.1562	0.033*	
C1S	0.02027 (7)	0.2996 (3)	0.05945 (7)	0.0298 (3)	
H1S1	−0.0177	0.2323	0.0396	0.045*	
H1S2	0.0544	0.2763	0.0275	0.045*	
H1S3	0.0134	0.4556	0.0653	0.045*	
C11	0.44173 (6)	0.8535 (3)	0.21911 (8)	0.0297 (3)	
H11	0.4776	0.9348	0.2082	0.036*	
O4S	0.15853 (5)	0.90631 (17)	0.46941 (5)	0.0285 (2)	
O3S	0.11677 (5)	0.61112 (17)	0.51804 (5)	0.0266 (2)	
C4S	0.16200 (7)	0.8869 (2)	0.59805 (7)	0.0292 (3)	
H4S1	0.1780	1.0354	0.5958	0.044*	
H4S2	0.1937	0.7926	0.6202	0.044*	
H4S3	0.1246	0.8849	0.6259	0.044*	
C3S	0.14591 (6)	0.8053 (2)	0.52335 (7)	0.0212 (3)	
C6S	0.44669 (7)	0.4352 (3)	0.45153 (8)	0.0347 (4)	
H6S1	0.4659	0.3259	0.4209	0.042*	
H6S2	0.4024	0.4505	0.4362	0.042*	
C5S	0.45086 (7)	0.3553 (3)	0.52918 (9)	0.0363 (4)	
H5S1	0.4317	0.2097	0.5322	0.044*	
H5S2	0.4274	0.4555	0.5591	0.044*	
C7S	0.48016 (7)	0.6561 (3)	0.44293 (9)	0.0370 (4)	
H7S1	0.4580	0.7693	0.4691	0.044*	
H7S2	0.4789	0.6974	0.3925	0.044*	

### Atomic displacement parameters (Å^2^)

**Table:**

	*U*^11^	*U*^22^	*U*^33^	*U*^12^	*U*^13^	*U*^23^
Cl	0.02984 (17)	0.0361 (2)	0.01704 (16)	0.00076 (14)	−0.00169 (12)	0.00358 (14)
O1	0.0221 (4)	0.0177 (4)	0.0275 (5)	−0.0006 (4)	−0.0010 (4)	0.0056 (4)
O2S	0.0311 (5)	0.0282 (5)	0.0183 (5)	0.0132 (4)	0.0001 (4)	0.0000 (4)
O1S	0.0258 (5)	0.0263 (5)	0.0179 (4)	0.0112 (4)	0.0001 (4)	0.0005 (4)
N3	0.0174 (5)	0.0156 (5)	0.0151 (5)	−0.0033 (4)	−0.0012 (4)	0.0029 (4)
N2	0.0154 (5)	0.0161 (5)	0.0165 (5)	−0.0018 (4)	0.0005 (4)	−0.0018 (4)
N1	0.0178 (5)	0.0222 (5)	0.0186 (5)	−0.0040 (4)	−0.0010 (4)	0.0033 (4)
C4	0.0183 (6)	0.0232 (7)	0.0175 (6)	−0.0036 (5)	−0.0034 (5)	0.0071 (5)
C6	0.0149 (5)	0.0185 (6)	0.0197 (6)	0.0015 (5)	−0.0005 (4)	−0.0006 (5)
C2S	0.0154 (5)	0.0197 (6)	0.0174 (6)	−0.0009 (5)	−0.0012 (4)	0.0017 (5)
C5	0.0164 (5)	0.0151 (6)	0.0192 (6)	−0.0001 (5)	−0.0024 (4)	0.0025 (5)
C2	0.0146 (5)	0.0179 (6)	0.0223 (6)	0.0017 (5)	−0.0010 (5)	0.0020 (5)
C8	0.0184 (6)	0.0274 (7)	0.0222 (6)	0.0019 (5)	−0.0036 (5)	−0.0073 (6)
C15	0.0201 (6)	0.0144 (6)	0.0155 (6)	0.0002 (5)	−0.0011 (4)	0.0011 (5)
C1	0.0133 (5)	0.0175 (6)	0.0196 (6)	0.0010 (4)	−0.0013 (4)	−0.0007 (5)
C17	0.0171 (5)	0.0184 (6)	0.0154 (6)	−0.0001 (5)	0.0010 (4)	0.0006 (5)
C7	0.0166 (6)	0.0259 (7)	0.0175 (6)	0.0040 (5)	−0.0005 (5)	0.0011 (5)
C9	0.0164 (6)	0.0192 (6)	0.0298 (7)	−0.0009 (5)	−0.0021 (5)	−0.0047 (5)
C16	0.0173 (6)	0.0141 (6)	0.0179 (6)	−0.0001 (4)	0.0003 (4)	−0.0003 (5)
C10	0.0266 (7)	0.0256 (7)	0.0264 (7)	−0.0086 (6)	−0.0048 (5)	0.0074 (6)
C3	0.0184 (6)	0.0235 (7)	0.0202 (6)	−0.0008 (5)	−0.0029 (5)	0.0074 (5)
C13	0.0228 (6)	0.0281 (7)	0.0193 (6)	−0.0038 (5)	−0.0009 (5)	0.0039 (6)
C14	0.0176 (5)	0.0173 (6)	0.0150 (6)	−0.0011 (5)	−0.0001 (4)	−0.0002 (5)
C12	0.0211 (6)	0.0394 (8)	0.0221 (7)	−0.0036 (6)	0.0017 (5)	0.0069 (6)
C1S	0.0350 (7)	0.0344 (8)	0.0197 (7)	0.0083 (6)	−0.0014 (6)	0.0068 (6)
C11	0.0221 (6)	0.0392 (8)	0.0278 (7)	−0.0122 (6)	−0.0008 (5)	0.0099 (6)
O4S	0.0308 (5)	0.0296 (5)	0.0254 (5)	0.0071 (4)	0.0062 (4)	0.0109 (4)
O3S	0.0309 (5)	0.0308 (5)	0.0182 (5)	−0.0071 (4)	0.0018 (4)	0.0010 (4)
C4S	0.0341 (7)	0.0292 (8)	0.0244 (7)	−0.0058 (6)	0.0035 (6)	−0.0020 (6)
C3S	0.0188 (6)	0.0233 (7)	0.0215 (6)	0.0054 (5)	0.0027 (5)	0.0031 (5)
C6S	0.0226 (7)	0.0481 (9)	0.0332 (8)	0.0057 (6)	−0.0017 (6)	−0.0085 (7)
C5S	0.0256 (7)	0.0440 (9)	0.0394 (9)	0.0011 (6)	0.0031 (6)	−0.0021 (7)
C7S	0.0302 (8)	0.0467 (9)	0.0341 (8)	0.0076 (7)	−0.0009 (6)	0.0030 (7)

### Geometric parameters (Å, º)

**Table:**

Cl—C7	1.7450 (13)	C16—H16A	0.9700
O1—C2	1.3936 (15)	C16—H16B	0.9700
O1—C3	1.3985 (16)	C10—C3	1.3856 (18)
O2S—C2S	1.2529 (15)	C10—C11	1.387 (2)
O1S—C2S	1.2623 (15)	C10—H10	0.9300
N3—C15	1.4918 (15)	C13—C12	1.3866 (18)
N3—C17	1.4919 (16)	C13—H13	0.9300
N3—H2N3	0.938 (17)	C14—H14A	0.9700
N3—H1N3	0.908 (16)	C14—H14B	0.9700
N2—C5	1.3916 (15)	C12—C11	1.388 (2)
N2—C14	1.4618 (15)	C12—H12	0.9300
N2—C16	1.4716 (15)	C1S—H1S1	0.9600
N1—C5	1.2863 (16)	C1S—H1S2	0.9600
N1—C4	1.4044 (16)	C1S—H1S3	0.9600
C4—C13	1.4006 (19)	C11—H11	0.9300
C4—C3	1.4010 (19)	O4S—C3S	1.2125 (16)
C6—C7	1.3852 (17)	O3S—C3S	1.3256 (16)
C6—C1	1.4005 (17)	O3S—H1S	0.94 (2)
C6—H6	0.9300	C4S—C3S	1.5019 (19)
C2S—C1S	1.5027 (17)	C4S—H4S1	0.9600
C5—C1	1.4905 (17)	C4S—H4S2	0.9600
C2—C9	1.3854 (18)	C4S—H4S3	0.9600
C2—C1	1.3929 (17)	C6S—C7S	1.520 (2)
C8—C9	1.3845 (19)	C6S—C5S	1.523 (2)
C8—C7	1.3867 (19)	C6S—H6S1	0.9700
C8—H8	0.9300	C6S—H6S2	0.9700
C15—C14	1.5156 (16)	C5S—C7S^i^	1.527 (2)
C15—H15A	0.9700	C5S—H5S1	0.9700
C15—H15B	0.9700	C5S—H5S2	0.9700
C17—C16	1.5164 (16)	C7S—C5S^i^	1.527 (2)
C17—H17A	0.9700	C7S—H7S1	0.9700
C17—H17B	0.9700	C7S—H7S2	0.9700
C9—H9	0.9300		
			
C2—O1—C3	111.72 (9)	C3—C10—C11	119.77 (13)
C15—N3—C17	110.86 (9)	C3—C10—H10	120.1
C15—N3—H2N3	110.0 (10)	C11—C10—H10	120.1
C17—N3—H2N3	111.0 (10)	C10—C3—O1	118.21 (12)
C15—N3—H1N3	109.7 (9)	C10—C3—C4	121.72 (13)
C17—N3—H1N3	110.1 (10)	O1—C3—C4	119.99 (11)
H2N3—N3—H1N3	105.1 (13)	C12—C13—C4	121.16 (13)
C5—N2—C14	116.76 (10)	C12—C13—H13	119.4
C5—N2—C16	117.53 (10)	C4—C13—H13	119.4
C14—N2—C16	112.18 (9)	N2—C14—C15	108.33 (9)
C5—N1—C4	123.41 (11)	N2—C14—H14A	110.0
C13—C4—C3	117.37 (12)	C15—C14—H14A	110.0
C13—C4—N1	117.62 (12)	N2—C14—H14B	110.0
C3—C4—N1	124.68 (12)	C15—C14—H14B	110.0
C7—C6—C1	119.10 (12)	H14A—C14—H14B	108.4
C7—C6—H6	120.4	C13—C12—C11	120.26 (13)
C1—C6—H6	120.4	C13—C12—H12	119.9
O2S—C2S—O1S	122.84 (11)	C11—C12—H12	119.9
O2S—C2S—C1S	119.38 (11)	C2S—C1S—H1S1	109.5
O1S—C2S—C1S	117.79 (11)	C2S—C1S—H1S2	109.5
N1—C5—N2	118.38 (11)	H1S1—C1S—H1S2	109.5
N1—C5—C1	126.41 (11)	C2S—C1S—H1S3	109.5
N2—C5—C1	114.71 (10)	H1S1—C1S—H1S3	109.5
C9—C2—C1	121.55 (12)	H1S2—C1S—H1S3	109.5
C9—C2—O1	118.71 (11)	C10—C11—C12	119.73 (13)
C1—C2—O1	119.72 (11)	C10—C11—H11	120.1
C9—C8—C7	119.01 (12)	C12—C11—H11	120.1
C9—C8—H8	120.5	C3S—O3S—H1S	110.1 (14)
C7—C8—H8	120.5	C3S—C4S—H4S1	109.5
N3—C15—C14	109.42 (10)	C3S—C4S—H4S2	109.5
N3—C15—H15A	109.8	H4S1—C4S—H4S2	109.5
C14—C15—H15A	109.8	C3S—C4S—H4S3	109.5
N3—C15—H15B	109.8	H4S1—C4S—H4S3	109.5
C14—C15—H15B	109.8	H4S2—C4S—H4S3	109.5
H15A—C15—H15B	108.2	O4S—C3S—O3S	119.90 (12)
C2—C1—C6	118.71 (11)	O4S—C3S—C4S	123.51 (13)
C2—C1—C5	121.06 (11)	O3S—C3S—C4S	116.59 (11)
C6—C1—C5	120.13 (11)	C7S—C6S—C5S	111.54 (13)
N3—C17—C16	109.77 (9)	C7S—C6S—H6S1	109.3
N3—C17—H17A	109.7	C5S—C6S—H6S1	109.3
C16—C17—H17A	109.7	C7S—C6S—H6S2	109.3
N3—C17—H17B	109.7	C5S—C6S—H6S2	109.3
C16—C17—H17B	109.7	H6S1—C6S—H6S2	108.0
H17A—C17—H17B	108.2	C6S—C5S—C7S^i^	110.96 (13)
C6—C7—C8	121.92 (12)	C6S—C5S—H5S1	109.4
C6—C7—Cl	118.96 (10)	C7S^i^—C5S—H5S1	109.4
C8—C7—Cl	119.13 (10)	C6S—C5S—H5S2	109.4
C8—C9—C2	119.70 (12)	C7S^i^—C5S—H5S2	109.4
C8—C9—H9	120.2	H5S1—C5S—H5S2	108.0
C2—C9—H9	120.2	C6S—C7S—C5S^i^	111.37 (13)
N2—C16—C17	110.55 (10)	C6S—C7S—H7S1	109.4
N2—C16—H16A	109.5	C5S^i^—C7S—H7S1	109.4
C17—C16—H16A	109.5	C6S—C7S—H7S2	109.4
N2—C16—H16B	109.5	C5S^i^—C7S—H7S2	109.4
C17—C16—H16B	109.5	H7S1—C7S—H7S2	108.0
H16A—C16—H16B	108.1		
			
C5—N1—C4—C13	148.72 (12)	C9—C8—C7—Cl	178.58 (9)
C5—N1—C4—C3	−38.18 (18)	C7—C8—C9—C2	0.34 (18)
C4—N1—C5—N2	−175.55 (11)	C1—C2—C9—C8	0.53 (18)
C4—N1—C5—C1	−4.1 (2)	O1—C2—C9—C8	178.70 (11)
C14—N2—C5—N1	10.69 (16)	C5—N2—C16—C17	−162.34 (10)
C16—N2—C5—N1	−127.00 (12)	C14—N2—C16—C17	58.13 (12)
C14—N2—C5—C1	−161.70 (10)	N3—C17—C16—N2	−54.59 (12)
C16—N2—C5—C1	60.61 (14)	C11—C10—C3—O1	−177.05 (11)
C3—O1—C2—C9	111.88 (12)	C11—C10—C3—C4	−0.4 (2)
C3—O1—C2—C1	−69.92 (13)	C2—O1—C3—C10	−117.96 (12)
C17—N3—C15—C14	−59.51 (12)	C2—O1—C3—C4	65.30 (14)
C9—C2—C1—C6	−0.46 (17)	C13—C4—C3—C10	0.51 (18)
O1—C2—C1—C6	−178.62 (10)	N1—C4—C3—C10	−172.60 (12)
C9—C2—C1—C5	−176.70 (11)	C13—C4—C3—O1	177.14 (11)
O1—C2—C1—C5	5.14 (17)	N1—C4—C3—O1	4.02 (18)
C7—C6—C1—C2	−0.46 (17)	C3—C4—C13—C12	0.09 (18)
C7—C6—C1—C5	175.81 (11)	N1—C4—C13—C12	173.70 (12)
N1—C5—C1—C2	38.92 (18)	C5—N2—C14—C15	159.85 (10)
N2—C5—C1—C2	−149.41 (11)	C16—N2—C14—C15	−60.29 (12)
N1—C5—C1—C6	−137.27 (13)	N3—C15—C14—N2	60.12 (12)
N2—C5—C1—C6	34.41 (16)	C4—C13—C12—C11	−0.8 (2)
C15—N3—C17—C16	56.29 (12)	C3—C10—C11—C12	−0.4 (2)
C1—C6—C7—C8	1.35 (18)	C13—C12—C11—C10	1.0 (2)
C1—C6—C7—Cl	−178.52 (9)	C7S—C6S—C5S—C7S^i^	−55.20 (19)
C9—C8—C7—C6	−1.29 (18)	C5S—C6S—C7S—C5S^i^	55.42 (18)

Symmetry code: (i) −*x*+1, −*y*+1, −*z*+1.

### Hydrogen-bond geometry (Å, º)

**Table:**

*D*—H···*A*	*D*—H	H···*A*	*D*···*A*	*D*—H···*A*
N3—H1*N*3···O1*S*	0.91 (2)	1.86 (2)	2.7664 (13)	175 (2)
O3*S*—H1*S*···O2*S*^ii^	0.94 (2)	1.61 (2)	2.5375 (13)	171 (2)
N3—H2*N*3···O1*S*^iii^	0.94 (2)	1.82 (2)	2.7292 (14)	162 (1)
C1*S*—H1*S*1···O3*S*^iii^	0.96	2.42	3.3778 (18)	172
C14—H14*A*···O1^iv^	0.97	2.59	3.2448 (15)	125
C17—H17*A*···O4*S*^iv^	0.97	2.32	3.2314 (15)	155

Symmetry codes: (ii) *x*, −*y*+1/2, *z*+1/2; (iii) −*x*, *y*−1/2, −*z*+1/2; (iv) *x*, *y*−1, *z*.
